# Consensual punishment does not promote cooperation in the six-person prisoner's dilemma game with noisy public monitoring

**DOI:** 10.1371/journal.pone.0188503

**Published:** 2017-11-27

**Authors:** Nynke van Miltenburg, Wojtek Przepiorka, Vincent Buskens

**Affiliations:** Department of Sociology / ICS, Utrecht University, Utrecht, The Netherlands; Middlesex University, UNITED KINGDOM

## Abstract

We study the effects of different punishment institutions on cooperation in a six-person prisoner’s dilemma game in which actors observe others’ cooperation with some noise (i.e. imperfect public monitoring). Previous research has shown that peer punishment can sustain cooperation, if a certain proportion of group members punish defectors at a cost to themselves. However, in the presence of noise, co-operators will sometimes be mistaken for defectors and punished, and defectors will sometimes be mistaken for co-operators and escape punishment. Both types of mistakes are detrimental for cooperation because cooperation is discouraged and defection is encouraged. By means of a laboratory experiment, we study whether this adverse effect of noise can be mitigated by consensual punishment. The more other group members have to agree on punishing a defector, the less likely will a co-operator be punished by mistake. We compare a punishment institution in which each subject decides individually whether to punish another, with institutions in which punishments are only implemented if subjects reach sufficient consensus that a particular group member should be punished. In conditions without noise, we find that cooperation and subjects’ payoffs are higher if more consensus is required before a punishment is implemented. In conditions with noise, cooperation is lower if more consensus is required. Moreover, with noise, subjects’ payoffs are lower under all punishment institutions than in the control condition without punishment opportunities. Our results narrow down the conditions under which punishment institutions can promote cooperation if such cooperation is noisy.

## Introduction

Many situations of human social interaction are characterized by a conflict between individual and collective interests. Prominent examples are cooperation problems in which actors decide on contributing their private resources to a collective endeavor. While full cooperation by all group members generates the best possible collective outcome, individual actors have an incentive to free ride on the contributions of others. This constitutes a social dilemma [1,2].

The question how the gap between individual and collective interests inherent in social dilemmas can be closed has been addressed by scholars from many disciplines [3,4,5,6,7]. It has been shown that repeated interactions among the same actors [8,9], the possibility for reputation formation [10,11,12], peer sanctioning [13,14], and institutions facilitating these mechanisms [15,16] can promote collectively beneficial outcomes in social dilemmas. Instigated by the success of lab experimental approaches to the studying of cooperation problems [17,18], peer-punishment has been controversially debated in the last 15 years [19].

Numerous studies of cooperation dilemmas consider settings in which, after observing the cooperation of their peers, actors can individually decide to reduce their peers’ payoffs at a cost to themselves. We call this a peer punishment institution that employs an ‘individual decision rule’ (henceforth IDR). In these studies, actors typically observe each other’s cooperation accurately and high cooperation rates are reached [13,20,21]. However, in numerous real world settings, individuals are provided with imprecise information about the cooperation or defection of others. Such situations are described as being subject to *imperfect public monitoring* or, simply, *noise* [22].

We study the effect of noise on cooperation under peer punishment institutions by means of a laboratory experiment with the six-person prisoner’s dilemma (PD) game. In the experimental conditions with noise, actors know that they observe other group members’ cooperation as defection (or vice versa) with a certain probability. The effect of noise on cooperation has been studied in the context of infinitely repeated PD games, in which cooperation can be sustained through direct reciprocity, if the probability of encountering the same actors again is large enough [23,24,25,26]. Here we study the effect of noise on the efficacy of peer-punishment institutions to promote cooperation in one-shot encounters.

It has been shown that noise can lead to misguided punishment decisions and thus limit the effectiveness of an IDR to sustain cooperation [27,28]. For example, let us assume that co-workers with different expertise work on a common project. While one of them, person A, may put little effort in the project, her co-workers, who know little about A’s field, may believe that she made a sincere contribution. Conversely, suppose person B offers valuable input that appears small, but which requires considerable work behind the scenes. Co-workers who did not observe B’s real effort may come to believe that B did not contribute his fair share. As the value of the common project depends on the actual contributions, all co-workers benefit from B’s but not from A’s effort. However, as some will misperceive A’s and/or B’s contributions, B may be criticized for free-riding, and A may not be sufficiently reprimanded for shirking. These sanctions or their absence, respectively, can occur even though A’s and B’s colleagues know that they might not observe A’s and B’s true efforts [27,28]. As a consequence, B may be discouraged from putting in the same amount of effort again, and A may be encouraged to continue shirking. In both cases, noise has a detrimental effect on cooperation.

An alternative peer punishment institution that might better support cooperation under noise is one that employs a collective decision rule (henceforth CDR), in which punishments are only carried out if a certain proportion of actors agrees to punish a particular group member. Numerous social groups that face cooperation problems employ collective decisions to implement punishment [16,29,30,31]. With noise, some peers correctly observe a cooperation, while others observe it as a defection and may punish accordingly. If more actors are required to agree, it becomes less likely that such ‘misguided’ punishments aimed at cooperators are implemented. At the same time, consensus on punishing true defectors might be difficult to reach, as one or several potential punishers may observe defectors as cooperators.

Hence, CDRs present both an advantage and a disadvantage with regard to implementing punishment in noisy environments. The magnitude of both effects depends on the required degree of consensus. Our aim is to identify an optimal decision rule for encouraging cooperation under noisy conditions—a rule that enables actors to identify and punish defectors, while cooperators are unlikely to be punished erroneously. In our experiment, we compare an IDR with two CDRs in series of one-shot six-person PD games with and without noise. The first CDR places minor restrictions on collective agreement, the second requires majority consensus. All conditions are preceded by a series of one-shot, six-person PD games without a peer-punishment opportunity, which we call the baseline condition.

In the remainder of this paper, we first summarize previous findings from related experimental studies. We then describe our experimental games, derive our hypotheses, describe the experimental design and procedure, and present our results. We conclude our paper with a discussion of our findings in the light of previous research.

## Related literature

In the abundant experimental literature on cooperation and peer punishment, a number of findings have been frequently replicated. In one-shot interactions without punishment options, cooperation rates are typically considerable, but depend on specific properties of the situation, exact payoffs, and characteristics of individuals [32]. If interactions are finitely repeated with changing partners, cooperation typically declines to lower levels over time [33,34]. Behaviors change considerably when peer punishment with an IDR is employed. Typically, high levels of cooperation are reached and maintained because many subjects punish defectors [20,21,35,36]. However, in recent years, evidence has accumulated that peer punishment with an IDR can also have detrimental effects [37,38]. For example, many studies find a small proportion of defectors who punish cooperators, which negatively affects cooperation [22,39,40]. Henceforth, we refer to punishment directed at defectors as *prosocial punishment*, because it is in the collective interest, and punishment directed at cooperators as *antisocial punishment* [40], because it is detrimental to collective interest. Moreover, since peer punishment is costly, many iterations are required before the benefits of increased cooperation outweigh punishment costs [41,42].

A growing body of research suggests that centralized sanctioning institutions have evolved to overcome the detrimental effects of peer punishment with an IDR [15,16,43,44]. For example, it has been argued that pool-punishment institutions, where free-riders are punished by a central authority that is maintained through voluntary contributions [45], have played an important role in maintaining cooperation at a large scale [46,47,48]. An important but understudied aspect in this strand of research is the procedure by which a central authority decides whom to punish, in particular, if cooperation cannot perfectly be observed [28]. Our study contributes to this strand of research by comparing the effectiveness of individual and collective decision rules for the implementation of punishment to maintain cooperation in noisy environments. Only a few studies have addressed closely related issues.

Two studies address peer punishment through CDRs. Casari and Luini [49] study groups of five subjects. Under their CDR, punishment is implemented if at least two subjects punish the same group member. The authors find that cooperation and earnings are higher under the CDR than under an IDR, as antisocial punishments hinder cooperation under the IDR but are not typically implemented under the CDR. Van Miltenburg et al. [50] examine groups of four actors. Two CDRs are employed: one for which two and one for which all three remaining group members must target the same recipient for punishment to be carried out. In this study, antisocial punishment is rare and does not affect cooperation even under the IDR. Moreover, prosocial punishments attempted under CDRs are often not implemented. Consequently, it is found that both contributions and earnings are lower when a broader consensus is required to enact punishments.

Both studies use the linear public goods game (PGG) in which subjects do not only decide whether to contribute, but also how much to contribute. We consider CDRs in a *n*-person PD setting where subjects merely decide whether to contribute their full endowment or nothing at all. Compared to experiments that use a linear PGG, our clear distinction between cooperation and defection may facilitate consensus on punishing defectors. At the same time, a linear PGG may facilitate consensus on punishing the lowest contributor [49], which is not possible in our setting if more than one actor defects.

Grechenig et al. [27] and Fischer et al. [28] experimentally examine how noise in the display of contributions affects the extent to which cooperation can be maintained through punishment institutions with an IDR. Both studies consider linear PGGs with noise. Noise is implemented as a 10% or a 50% probability of a contribution to be observed as a random amount. This renders noise more ambiguous than it is in our setting, in which a misrepresented contribution is always shown as defection and vice versa. Grechenig et al. [27] and Fischer et al. [28] find that subjects do not refrain from employing punishment in the presence of noise, such that two types of errors occur in prosocial punishment. First, some punishments are mistakenly directed at cooperators. Whereas Grechenig et al. [27] show that mistaken punishment of cooperators is detrimental to cooperation, Fischer et al. [28] cannot confirm this result. Second, defectors evade punishment from others who observe them as cooperators and are thus less strongly discouraged from free riding. Moreover, resources are ‘wasted’ when punishments do not reach the intended target. As a result, both studies find that in the presence of noise, an IDR cannot promote cooperation and earnings as effectively as without noise. This latter finding is supported by studies which implement noise or inaccurate information about actors’ contributions in a way that is less related to our setup [41,51,52].

In a recent working paper, Ambrus and Greiner [53] report the results of an experiment that seems closest to ours. They also investigate the effectiveness of a CDR to promote cooperation in an *n*-person PD with noisy public monitoring. They find that the CDR is more successful in maintaining cooperation than the IDR both with and without noise. Since their findings stand in stark contrast to ours, we will discuss in detail possible explanations for this difference in the last section of our paper.

## Experimental game and hypotheses

### One-shot n-person PD with peer punishment

We consider cooperation problems in series of one-shot six-person PDs [54,55,56]. The PD model is employed due to the straightforward manner in which noise can be incorporated. In a six-person PD, all *n* = 6 actors *i* receive an equal endowment *w*. All actors independently and simultaneously determine whether to contribute their entire endowment to a group project, i.e., contribution *c*_*i*_ is either 0 (defection) or *w* (cooperation). All contributions *c* = ∑*c*_*i*_ are multiplied by *m*, with 1 < *m* < *n*, and divided equally among all members. As *m* < *n*, cooperation generates a lower payoff than defection (*wm*/*n* < *w*). However, group payoffs (*nw*–*c* + *mc*) are maximized if all actors cooperate. Moreover, under full cooperation, individuals earn higher payoffs than they do under full defection (*wm* > *w*). Individually rational and selfish behavior thus leads to Pareto-suboptimal outcomes, rendering the one-shot PD a classic example of a social dilemma [1,2]. In our experiment, we use common values for endowments and individual returns from contributing by setting *w* = 20 and *m* = 2.4 [20,21].

We focus on PDs with peer punishment opportunities. Following the contribution stage described above, each actor *i* observes the contribution decisions of all other group members *j* ≠ *i*. All actors then individually and simultaneously determine whether to punish each *j*. If *i* decides to punish *j*, and if the punishment is implemented, actor *i* pays a fixed cost of *a* > 0, while *j* loses an amount of *b* > *a*. If *i* decides not to punish *j*, actor *i* pays no cost, and the earnings of *j* are unaffected. The total number of group members that *i* allocates punishment to is denoted by *f*_*i*_; the total number of group members who punish *i* is denoted by *g*_*i*_. In the experiment, we employ *a* = 2 and *b* = 6, which corresponds to the frequently used 1:3 cost-to-impact ratio of punishment [57].

Under an IDR, which reflects how peer punishment institutions are typically employed in cooperation experiments, all punishments are implemented. Thus, each actor’s earnings are decreased by allocated punishments *af*_*i*_ and received punishments *bg*_*i*_. Under a CDR, punishment is only implemented if at least a certain proportion *x*/(*n–* 1) of group members *j* ≠ *i* punishes the same actor *i*. If *g*_*i*_*/*(*n–* 1) < *x/*(*n–* 1), none of the punishments directed at *i* are carried out, i.e., actors *j* do not incur cost *a* for punishing *i*, and *i*’s earnings are not reduced. Thus, an actor *i* only loses an amount of *bg*_*i*_ due to punishments received if *g*_*i*_*/*(*n–* 1) ≥ *x/*(*n–* 1), and only incurs punishment costs of *a* for each *j* whom *i* has attempted to punish *and* for whom *g*_*j*_*/*(*n–* 1) ≥ *x/*(*n–* 1). Actors are not informed of non-implemented punishments that others attempt to allocate.

In our experiment, we employ two different CDRs: one under which punishment is implemented if at least two actors are willing to punish the same recipient (CDR2), and one under which at least three punishers should agree on punishing (CDR3). In our groups of six, up to five group members can punish each actor. Thus, CDR3 requires a majority for punishment to be carried out (*x*/(*n–* 1) = 0.6), while CDR2 requires the lowest possible degree of consensus (*x*/(*n–* 1) = 0.4).

Under an IDR, rational and self-regarding actors who assume that others are also rational and self-regarding will not allocate or expect to receive punishment in (a series of) one-shot interactions, as opportunities for reputation building are ruled out. Under CDRs, rational, self-regarding actors likewise do not punish others if the punishment is implemented. If a punishment is not implemented, actors are indifferent toward punishing or not. The unique subgame-perfect Nash equilibrium of zero contributions of the baseline PD remains unchanged, but punishing below the required level of consensus is allowed in equilibrium.

### Noise in the display of contributions

In most experiments using voluntary contribution games (e.g., PGG or PD), subjects receive accurate information on the contribution decisions of all other group members. Here, we compare this standard setup with one in which actors know that there is a 20% probability that they observe another group member’s decision to cooperate as defection, or a defection as cooperation. Whether another group member’s contribution decision is displayed correctly is independently determined for each actor. Hence, on average, each actor’s contribution decision will be incorrectly perceived by one of the five other group members. However, payoffs are based on the real contributions of all group members.

Assuming that initial cooperation rates will be close to 50%, as is frequently found in linear PGG experiments [33,34], observed initial cooperation rates should not be much affected by noise because an approximately equal number of contributions and defections will be perceived incorrectly. Thus, a typical decline in cooperation in the PD without punishment should also occur in the presence of noise. Hence, we refer to the baseline PD as a PD without punishment institution, and assume that it does not matter whether noise is present or not.

With regard to peer punishment, there are two ways in which noise can cause actors’ actions to deviate from their intentions (henceforth referred to as *punishment errors*). First, prosocial punishers might fail to punish actual defectors if they observe these defectors as cooperators. Second, prosocial punishers may punish actual cooperators if they observe these cooperators as defectors. This latter punishment error is different from antisocial punishment, which is aimed at actual cooperators [40]. The two punishment errors change the amount of punishment that cooperators and defectors can expect to receive.

Table 1 lists the punishments that defectors and cooperators can expect to receive in our experiment given the number of other group members who punish observed defectors. For example, an actual cooperator might face three group members who punish observed defectors and two others who never punish. Without noise, the cooperator will not be punished, irrespective of the decision rule. With noise, each of the three potential punishers might observe the cooperator as a defector and attempt to punish. Now the expected amount of punishment also depends on the decision rule. Under CDR3, a punishment is only implemented if all three potential punishers observe the cooperator as a defector and attempt to punish. This is relatively unlikely (0.2×0.2×0.2 = 0.008). If only one or two punishers observe the wrong contribution decision under CDR3, the cooperator is not punished. Under an IDR, the likelihood to be punished by at least one group member is relatively high (1 − 0.8×0.8×0.8 = 0.488). Note that in the same way as CDRs filter out punishment errors, they filter out anti-social punishment [49], and a related study has identified the latter as an important driver of cooperation [53]. However, since in our experiment the occurrence of antisocial punishment is very low, we refrain from theorizing about it further.

**Table 1:** Punishment points for cooperators and defectors, and the difference in punishment points between cooperators and defectors with noise, for each experimental condition. Each row corresponds to a different number of prosocial punishers in a group. Antisocial punishment is not considered. For noise conditions, the table shows expected values based on an average of 20% inaccurate observations. Values are based on parameters used in the experiment.

Number of prosocial punishers	No noise						Noise								
	IDR		CDR2		CDR3		IDR			CDR2			CDR3		
	D	C	D	C	D	C	D	C	diff.	D	C	diff.	D	C	diff.
1	6	0	0	0	0	0	4.80	1.20	3.60	0	0	0	0	0	0
2	12	0	12	0	0	0	9.60	2.40	7.20	7.68	0.48	7.20	0	0	0
3	18	0	18	0	18	0	14.40*	3.60	10.80	13.82^ǂ^	1.30	12.53	9.22	0.14	9.07
4	24	0	24	0	24	0	19.20	4.80	14.40	19.05	2.34	16.70	17.20	0.50	16.70
5	30	0	30	0	30	0	24.00	6.00	18.00	23.98	3.52	20.46	23.37	1.06	22.31

* Example calculation: the probability that zero, one, two or three prosocial punishers observe a defection multiplied by associated punishment points gives 0.008×0 + 0.096×6 + 0.384×12 + 0.512×18 = 14.40.

^ǂ^ Example calculation: if only one out of three prosocial punishers correctly observes a defection, punishment is not implemented under CDR2. Expected punishment is thus calculated as follows: (0.008+0.096)×0 + 0.384×12 + 0.512×18 = 13.82.

The amount of punishment an actual defector can expect is calculated accordingly. Without noise and under IDR, punishment for defection is the number of punishers multiplied by the points that recipients lose for each punishment (i.e. *bg*_*i*_). For example, defectors who are punished by three group members lose 3×6 = 18 points. In CDR conditions, defectors receive no punishment if the number of punishers falls below the implementation threshold. With noise, punishment depends on the number of punishers who correctly observe a defection. Thus, if a group has *k* punishers, the probability that zero, one, …, *k* of these punishers observe a defection correctly is used to weigh the corresponding punishment level. For an actual defector under the IDR with three punishers, there is a 0.8% chance that no punisher correctly observes the decision (0.2×0.2×0.2 = 0.008) and the defector receives no punishment at all. Likewise, there is a 9.6% chance that only one punisher observes the defector correctly (3×0.2×0.2×0.8 = 0.096), causing the defector to receive six punishment points, etc. (see the example in the Table 1 notes). Under a CDR, even if enough punishers are available, punishment is only implemented if sufficient punishers correctly observe the defection. Thus, the same probabilities for any number of punishers correctly observing a defection apply as under the IDR, but zero punishment points are associated with cases in which too few punishers observe the defection (see Table 1).

### Hypotheses

We use Table 1 to derive hypotheses regarding differences in cooperation rates and earnings across experimental conditions. We predict that cooperation rates will be higher the more punishment defectors receive relative to cooperators. We assume risk neutral actors and base our hypotheses on two further assumptions: (1) punishment directed at cooperators reduces cooperation to the same extent as punishment directed at defectors promotes cooperation, and (2) this effect of punishment on cooperation remains the same irrespective of whether contribution decisions are observed with or without noise. We test the last two assumptions empirically with our experimental data.

Without noise, we have no reason to assume that punishment decision rules affect actors’ propensity to punish defectors. As non-implemented punishments under a CDR are costless and not communicated to other group members, actors have no incentive to withhold punishment even if they believe that not enough other group members will propose to punish the same actor. However, this is not to say that decision rules have no bearing on the amount of implemented punishment. Table 1 shows that without noise, fewer prosocial punishments are implemented when decision rules are stricter. Thus, assuming the absence of antisocial punishment, cooperation rates should be higher when fewer actors are required to agree on punishment decisions.

Noise might render actors reluctant to punish observed defectors, as they may punish an actual cooperator [51,52]. However, the more consensus is required, the lower the probability of punishment errors, and the more closely punishment decisions should correspond to the situation without noise. Thus, we expect that actors will be less likely to punish under noise than without noise, but more likely to punish under noise the more consensus is required.

For any number of punishers, Table 1 shows that the amount of punishment cooperators can expect is higher and the amount of punishment defectors can expect is lower with noise than without noise, irrespective of the punishment decision rule. This effect of noise should negatively affect cooperation. With regard to earnings, if cooperation rates increase as a result of prosocial punishment, without noise, this implies that fewer punishment costs must be paid. Conversely, with noise even when full cooperation is achieved, some actors are observed as defectors and may be punished [41]. Thus, higher punishment costs must be paid if noise is present, and these punishment costs are offset by a smaller increase in cooperation. Accordingly, we expect noise to negatively affect both cooperation and earnings:

**Hypothesis 1a**: With a punishment institution, less cooperation is achieved with noise than without noise regardless of the punishment decision rule employed.

**Hypothesis 1b**: With a punishment institution, lower earnings are achieved with noise than without noise regardless of the punishment decision rule employed.

We expect that the decision rule resulting in the highest punishment of defectors relative to cooperators will generate the highest cooperation rates. The shaded cells in Table 1 highlight the decision rules that generate the highest difference between the amount of punishment cooperators and defectors can expect from a given number of prosocial punishers. For example, if five prosocial punishers are present, the difference between expected punishment for cooperation and for defection is highest under CDR3 (22.3 points). In case of two, three or four punishers, CDR2 yields the largest differences. Moreover, if a CDR renders actors more likely to punish with noise, such that more prosocial punishments are allocated under CDR2 than under the IDR, the difference in cooperation between CDR2 and the IDR may be even stronger. If actors are even more likely to punish under CDR3 than under CDR2, the difference in cooperation between CDR2 and CDR3 may be less pronounced. Assuming an intermediate number of punishers (two, three or four out of five), we formulate the following hypothesis:

**Hypothesis 2a**: With noise, cooperation rates are higher under CDR2 than under the IDR and CDR3.

Under CDR2, for each given number of punishers in a group, lower punishment of both cooperators and defectors is expected than under the IDR. Since we hypothesize that higher cooperation rates are achieved under CDR2 than under the IDR, and that fewer punishments are allocated to achieve this, we can also expect that earnings will be higher under CDR2 than under the IDR.

**Hypothesis 2b**: With noise, earnings are higher under CDR2 than under the IDR.

We refrain from comparing CDR2 and CDR3 under noise in terms of earnings; the lower cooperation rates we expect under CDR3 reduce earnings but, at the same time, fewer punishments are implemented under CDR3, which reduces the costs. The net effect of CDRs on earnings is difficult to predict under noise.

We assume risk neutrality to be able to formally calculate expected payoffs and to illustrate how expected punishments vary with numbers of punishers in Table 1. In the conditions with noise, defectors face a lower and cooperators face a higher risk of being punished than without noise. This implies that if we assume risk-averse actors, our hypotheses are reinforced. Moreover, since with noise risks of being punished are relatively comparable across decision rules, our current hypotheses hold for risk-averse actors in this regard as well.

## Materials and methods

### Ethics statement

This research was reviewed and approved by the Ethics Review Board of the Faculty of Social and Behavioural Sciences of Utrecht University. The experiment was conducted in accordance with ethical guidelines of the Experimental Laboratory for Sociology and Economics (ELSE) at Utrecht University (https://www.elseutrecht.nl). All subjects had given written informed consent before participating in our experiment. The anonymized data are available online as Supporting Information to this paper (S1 Data).

#### Experimental design and procedures

The experiment was programmed in z-Tree [58] and conducted at the Experimental Laboratory for Sociology and Economics (ELSE) at Utrecht University. Subjects were recruited through ORSEE [59]. A total of 252 subjects participated in the experiment (38% male, 86% students, 32% economics students, average age of 22.57). The number of subjects per session was either 18 or 24 and a session lasted one hour on average. A participant’s earnings averaged €11, with a minimum of €7 and a maximum of €14.

We conducted twelve sessions, six with noise and six without noise (see Table 2). In each session, subjects participated in three sequences of 15 six-person PD games (i.e. 45 periods in total). In each round, every subject was endowed with *w* = 20 points and could decide whether or not to contribute the entire endowment. The total amount contributed in each round was multiplied with *m* = 2.4 and equally divided among the six members of a group (see the Experimental Game section for details on the PD game). The groups of six subjects were disbanded and randomly formed anew after each round (i.e. random matching). Given the number of participants per session, under this matching scheme it is likely that subjects interact with the same partners multiple times. We account for such interdependencies within sessions in our analyses.

**Table 2:** Number of subjects per experimental session.

Decision rules in second and third sequence	without noise (# subjects)	with noise (# subjects)
IDR—CDR2	18	18
IDR—CDR3	24	24
CDR2—CDR3	18	18
CDR2—IDR	24	24
CDR3—IDR	18	24
CDR3—CDR2	18	24

*Notes*: We conducted 12 sessions with 18 or 24 participants in each session. Each session started with a sequence of 15 periods without punishment and was followed by two sequences of 15 periods with a punishment stage. The punishment decision rule was varied across the second and third sequence.

Subjects first participated in a sequence of 15 PD games without the option to punish other group members. In sessions without noise, subjects were perfectly informed of the contribution decisions of others and of their own earnings after each game. In sessions with noise, subjects were only informed of noisy contribution decisions and corresponding earnings. However, they were made aware that they observed each contribution decision and corresponding earnings incorrectly with a probability of 20%. An incorrect observation implied that an actual cooperation was displayed as a defection and vice versa.

After the first sequence, a punishment stage was added to every PD game for the two ensuing sequences of 15 periods. In each period, after being informed about the (noisy) contributions of others in the PD, all subjects received an additional endowment of 10 points and decided for each of the other group members whether or not to punish them. If a subject decided to punish and the punishment was implemented, six points were deducted from the earnings of the recipient, and two points were deducted from the earnings of the punishing subject. The additional endowment of 10 points thus enabled each subject to punish each of the five other group members. Whether or not subjects receive an extra punishment endowment does not seem to affect punishment decisions [27]. When multiple subjects targeted the same group member with punishment and the punishment was implemented, all punishers paid the punishment cost, and the targeted subject was deducted the cumulative amount. For example, a subject who was punished by four others lost 24 points.

As noted above, three punishment decision rules were employed as experimental conditions. In each session, the two sequences of 15 periods with punishment opportunity were each conducted under a different decision rule (see Table 2). Under the IDR, all punishments that subjects proposed were implemented. Under CDR2, punishments were only implemented if at least two group members were willing to punish the same recipient. Under CDR3, at least three punishers were required for a punishment to be implemented. Further information on punishment implementation through different decision rules is provided in the Experimental Game section.

After the punishment stage, subjects were shown a screen with others’ (noisy) contribution decisions, in the same way they saw them after the contribution stage, and with implemented punishments that each group member had received. Participants were not informed about non-implemented punishments, and could not infer who had allocated the punishments. Again, in the sessions without noise, subjects were informed of their actual earnings after each period, while in the sessions with noise, subjects were informed of the payoff they would have received if their observed contribution decisions were actual decisions. Only at the end of the experiment, all subjects were informed of their actual aggregate earnings. Subjects received €1 for every 160 points they earned in the experiment.

Note that subjects’ behavior that occurred in the first sequence of 15 rounds with punishment opportunity remained largely consistent throughout the second sequence despite changes in decision rules (see Fig A in S1 Additional analyses and study material). A Fisher’s exact test confirms that sessions in which cooperation rates are above average in the first sequence tend to generate above-average cooperation rates in the second sequence (*p* = 0.08). In the Results section, we therefore only report results of the first sequence of PD games with a punishment stage. More detailed analyses of the results of the second punishment sequence are available from the authors on request.

## Results

### Cooperation rates

**Fig 1:**
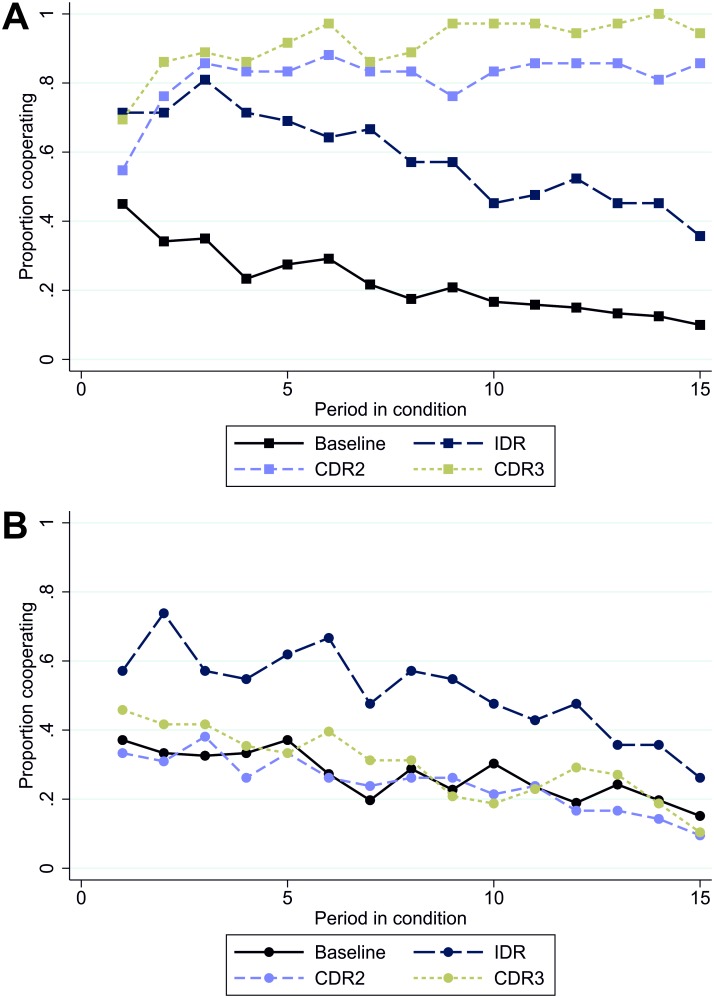
Cooperation in each period of the baseline and first punishment sequence by experimental condition without (panel A) and with (panel B) noise.

Fig 1 presents the proportion of subjects who cooperate in the PD over time and across experimental conditions. In panel A, it is evident that in the absence of noise, all decision rules lead to an increase in cooperation rates relative to the baseline condition without a punishment stage. Additionally, cooperation rates increase as more group members are required to agree on punishment decisions. Only under the CDRs cooperation is maintained at a high level. This contradicts the typical experimental finding that an IDR without noise generates increasing cooperation rates [35,36]. Panel B of Fig 1 presents a very different picture. With noise, only the IDR has a cooperation-enhancing effect relative to the baseline condition, which weakens over time. Cooperation rates for both CDRs are very similar to the baseline condition.

### Test of hypotheses on cooperation

Differences between the conditions shown in Fig 1 are confirmed through a regression analysis. Throughout the paper, we present results based on multilevel regression models with random effects at subject and session levels to control for interdependencies we expect at both levels. The interdependencies at the session level are due to using random matching. We verify the robustness of our estimates in regression models with observations clustered at the session level also based on bootstrap estimation (see Tables C, D and E in S1 Additional analyses and study material). Because there were only 12 experimental sessions, clustering at session level generates very conservative estimates. Nevertheless, models with a session-level cluster support our main conclusions. We report below if a hypothesized effect is not robust in models with session-level clustering.

**Fig 2:**
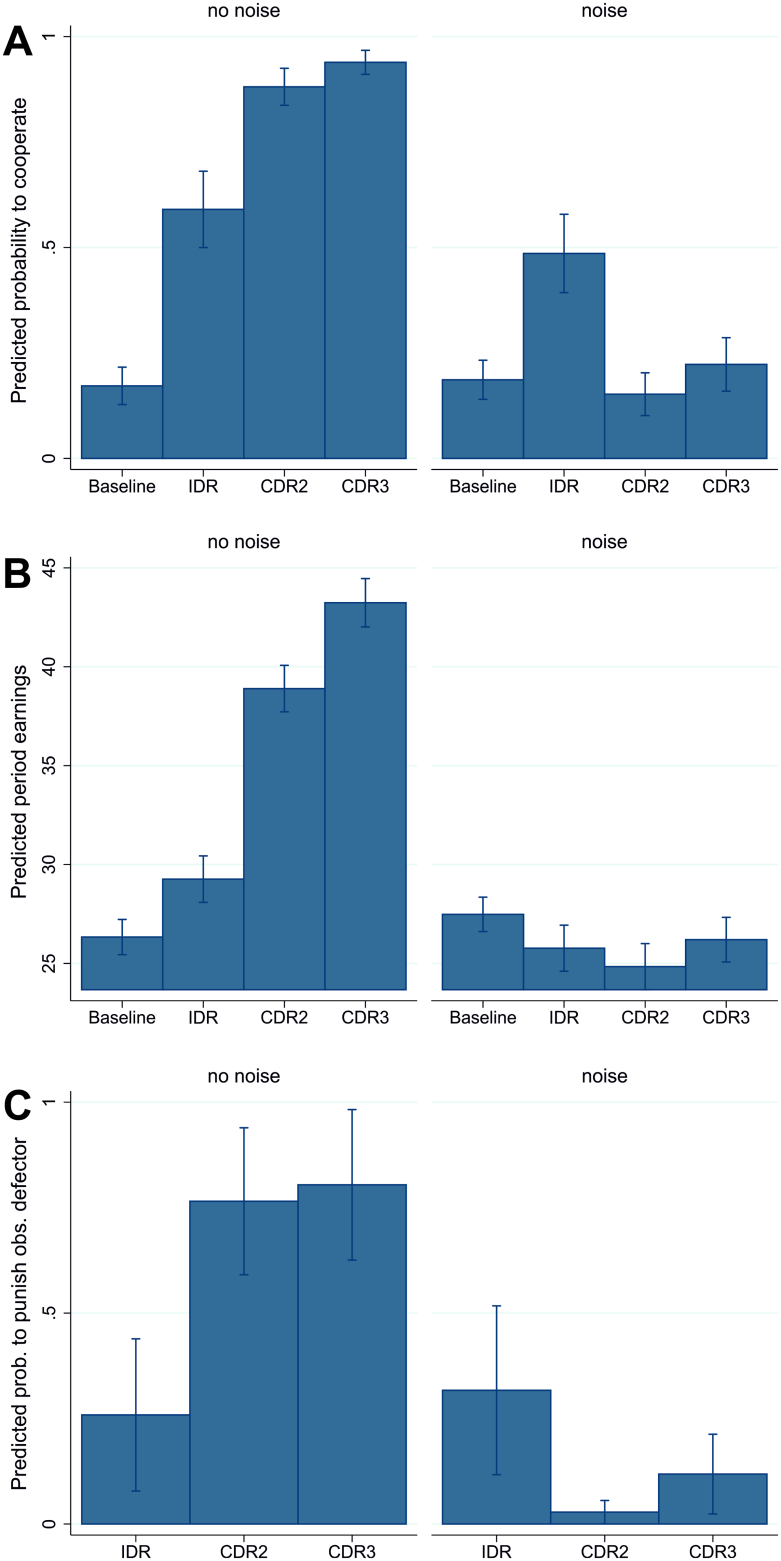
Predicted probability to cooperate (panel A), predicted earnings (panel B; excluding punishment endowment), and predicted probability to punish an observed defector (panel C) across experimental condition, with 95% confidence intervals. These predictions are based on regression models shown in Table B in the S1 Additional analyses and study material.

Panel A of Fig 2 shows the predicted probability that a subject cooperates for each experimental condition. These predictions are based on the multilevel regression model presented in Table B in S1 Additional analyses and study material (the corresponding descriptive statistics are listed in Table A in S1 Additional analyses and study material). The “Cooperation” column in Table 3 lists the differences in these predicted probabilities across experimental conditions (the predicted probabilities and significance of the differences are calculated using *margins* and *pwcompare* in STATA 13 after the estimation of the multilevel logit). For example, our model predicts a 0.014-point higher probability to cooperate with noise than without noise in the baseline games. This difference is not significant.

**Table 3:** Differences between experimental conditions of predicted cooperation probability, predicted earnings (excluding punishment endowment), and predicted probability to punish an observed defector. Based on the fixed segments of multilevel logistic (cooperation and punishment) and linear (earnings) regression models with decisions nested in subjects and sessions. The actual models are displayed in Table B in S1 Additional analyses and study material (7,955 punishment decisions, 7,560 PDs, 252 subjects).

	Cooperation		Earnings		Punishment obs. defectors	
	Diff.	S.e.	Diff.	S.e.	Diff.	S.e.
*Noise vs*. *no noise*						
Baseline	0.014	0.033	1.146	0.635		
IDR	-0.104	0.066	-3.493**	0.844	0.058	0.137
CDR2	-0.729**	0.035	-14.059**	0.844	-0.737**	0.090
CDR3	-0.716**	0.035	-17.039**	0.848	-0.686**	0.104
*Without noise*						
IDR—Baseline	0.418**	0.035	2.928**	0.492		
CDR2—Baseline	0.709**	0.021	12.563**	0.492		
CDR3—Baseline	0.767**	0.020	16.906**	0.529		
CDR2—IDR	0.291**	0.042	9.635**	0.682	0.507**	0.128
CDR3—IDR	0.349**	0.044	13.978**	0.708	0.546**	0.130
CDR3—CDR2	0.058*	0.023	4.343**	0.708	0.039	0.126
*With noise*						
IDR—Baseline	0.300**	0.035	-1.711**	0.492		
CDR2—Baseline	-0.034	0.020	-2.643**	0.492		
CDR3—Baseline	0.037	0.023	-1.279**	0.462		
CDR2—IDR	-0.334**	0.042	-0.931	0.682	-0.289**	0.103
CDR3—IDR	-0.263**	0.042	0.432	0.662	-0.199	0.113
CDR3—CDR2	0.071*	0.030	1.363*	0.662	0.090	0.050

*Significant at .05-level;

** Significant at .01-level (2-sided)

We expected that noise is detrimental for cooperation under each decision rule (Hypothesis 1a). Table 3 shows no significant difference in the predicted probability to cooperate between the noise and the no noise condition under the IDR. However, predicted probabilities to cooperate are significantly lower with noise than without noise under CDR2 and CDR3. These results support our hypothesis for both CDRs but not for the IDR. We also expected that CDR2 would lead to the highest cooperation levels under noise (Hypothesis 2a). However, with noise only the IDR generates a significantly higher predicted cooperation probability than the noise baseline. The predicted probability to cooperate is significantly higher under the IDR than CDR3, and significantly higher under CDR3 than CDR2. Thus, Hypothesis 2a is not supported. The difference between the two CDRs disappears in the model with clustering at the session level (see S1 Additional analyses and study material).

### Test of hypotheses on earnings

Panel B of Fig 2 shows predicted period earnings for each experimental condition. Descriptive statistics are provided in Table 1 and predictions are derived from the multilevel regression model presented in Table B in S1 Additional analyses and study material. The”Earnings” column in Table 3 lists differences in predicted earnings across experimental conditions. The results are in accordance with those for the differences in predicted cooperation probabilities. Predicted earnings are significantly lower with noise than without noise under all decision rules, supporting Hypothesis 1b. With noise, predicted earnings fall significantly below the baseline levels under all decision rules. The differences between the IDR and the CDRs are insignificant. However predicted earnings are higher under CDR3 than under CDR2. Thus, our hypothesis that CDR2 would generate the higher earnings than the IDR under noise (Hypothesis 2b) is not supported. The difference in predicted earnings between the CDRs disappears if session clustering is accounted for in the estimation (see S1 Additional analyses and study material).

To explain the effects of the experimental conditions on cooperation and earnings that do not support our hypotheses, we now present results on punishment behavior and a detailed analysis of how punishment affects subsequent contribution decisions.

### Test of assumptions—Punishment decisions

Panel C of Fig 2 shows the predicted probabilities to punish an observed defector for each experimental condition (the probabilities to punish observed cooperators are very low and do not differ across experimental conditions; we therefore refrain from reporting these results here). Descriptive statistics are listed in Table A and the multilevel regression model on which the predictions are based are presented in Table B in S1 Additional analyses and study material. In each period, subjects observed between zero and five defectors in their group, for whom they decide whether or not to punish. The “Punishment obs. defectors” column in Table 3 lists the differences between predicted punishment probabilities across experimental conditions.

We expected that decision rules do not affect punishment decisions without noise, that actors might be less likely to punish with noise than without noise, and that actors would more likely punish with noise as more agreement is required. Table 3 shows that predicted punishment probabilities are indeed significantly lower with noise than without noise under CDR2 and CDR3. However, without noise, relative to the IDR, the predicted probabilities to punish are significantly higher under CDR2 and CDR3, while with noise, the predicted probability to punish is significantly lower under CDR2 than under the IDR. Thus, with noise, actors are *less* likely to punish when the likelihood that prosocial punishment errors are implemented is lower, and the effect of required collective agreement on the likelihood to punish differs between the two noise conditions. This is inconsistent with the assumptions underlying our hypotheses. To examine how these observations affect cooperation, we next analyze the effect of receiving punishment on subsequent contribution decisions.

### Test of assumptions—Previous game effects on contribution decisions

Table 4 presents a regression model with subjects’ contribution decisions as binary dependent variable. Subjects’ own previous contribution decision, received punishments, the number of other group members that a subject observed as cooperators in the previous period, and noise interactions for these variables are included as explanatory variables in the model. Punishments received for contributing and for defecting are included as two separate dummy variables. Models with a continuous variable indicating by how many others a subject was punished suffer from multi-collinearity issues in the conditions without noise because the number of punishers is highly correlated with the punished subject’s and the other group members’ decisions to cooperate. These models are therefore not considered here.

**Table 4:** Multilevel logistic regression on cooperation decisions for period t, with decisions nested in subjects and sessions in the first punishment sequence (3,528 PDs, 252 subjects).

	Coeff.	S.e.
**Main effects**		
Noise	1.157	0.652
CDR2	1.234**	0.441
CDR3	1.954**	0.502
Own contribution *t* − 1	2.766**	0.450
Punished while defecting *t* − 1	1.276*	0.511
Punished while cooperating *t* − 1	-0.863*	0.365
Obs. *N* other cooperators *t* − 1	0.416**	0.085
Period	-0.041*	0.020
**Interaction effects with noise**		
CDR2	-2.784**	0.636
CDR3	-2.986**	0.660
Own contribution *t* − 1	-1.924**	0.533
Punished while defecting *t* − 1	-0.699	0.561
Punished while cooperating *t* − 1	1.074*	0.449
Obs. *N* other cooperators *t* − 1	-0.208*	0.107
Period	-0.098**	0.027
Constant	-2.471**	0.612
σ_u_	0.000	0.353
σ_e_	1.769**	0.143
Log likelihood	-1407.135	

* Significant at the .05-level;

** Significant at the .01-level (two-sided)

Table 4 shows that subjects are significantly more likely to cooperate when they had cooperated previously. With noise, this effect is still significant but half as strong, reflecting higher fluctuations in cooperation over time. Receiving punishment for defection has a significantly positive effect on subsequent cooperation both without and with noise. The latter is established by means of a Wald test for linear hypotheses, adding the corresponding main and the interaction terms listed in Table 4 (1.276–0.699 = 0.577: χ^2^_(1)_ = 6.27, *p* = 0.012). Receiving punishment for cooperation has a significantly negative effect on subsequent cooperation in the no-noise conditions only; adding the corresponding main and interaction terms listed in Table 4 results in an insignificant test statistic (-0.863 + 1.074 = 0.211: χ^2^_(1)_ = 0.65, *p* = 0.421). Hence, our assumption that punishment of defectors is as beneficial to cooperation as punishment of cooperators is harmful is not confirmed for the noise conditions. Additional analyses show that punishment received for defection does have a highly significant effect with noise if specified as a continuous variable. The effect of punishment received for cooperation is insignificant regardless of precise specifications. Finally, the more other group members were observed as cooperators in the previous interaction, the higher the likelihood of subsequent cooperation. This effect is significant but much weaker in the noise conditions.

In sum, the high prosocial punishment levels under CDR2 and CDR3 without noise significantly increase subsequent cooperation, while the less frequent prosocial punishment under noise did not have a significant effect. This explains why, contrary to our expectations, cooperation is not supported under CDRs with noise.

## Discussion and conclusions

In our experiment, we compare the effect of different decision rules for implementing punishment on cooperation and earnings in series of one-shot six-person PDs with and without noise in the display of contribution decisions. We hypothesize that cooperation rates are lower with noise than without noise under each decision rule (Hypothesis 1a). This hypothesis is supported for the collective decision rules (CDRs), but not for the individual decision rule (IDR). The cooperation rate under the IDR without noise is surprisingly low and not different from the corresponding rate in the condition with noise. Under all decision rules, we find earnings to be negatively affected by noise. This supports our Hypothesis 1b. Furthermore, we hypothesize that cooperation rates in the noise conditions are higher under the least restrictive CDR (CDR2) than under a decision rule that requires majority consensus (CDR3) and the IDR (Hypothesis 2a). We also predicted earnings to be higher under CDR2 than under the IDR (Hypothesis 2b). These hypotheses are not supported. Instead, with noise cooperation rates are higher under the IDR than under both CDRs, and there is slightly more cooperation under CDR3 than CDR2. We find no differences in earnings between the IDR and CDR2, while earnings under CDR3 are higher than in the other two conditions. Moreover, earnings under all decision rules that include noise are lower than in the baseline condition without punishment possibility.

Noise effects and differences between decision rules that we find at the macro level can partly be explained by noise effects on behaviors at the micro level. First, with noise, we find that subjects are less likely to punish observed defectors under the CDRs than the IDR. Second, punishment directed at cooperators is detrimental to cooperation without noise, but does not significantly affect cooperation with noise. Punishment directed at defectors positively affects cooperation regardless of noise. These two results imply that if noise is present, only under the IDR punishment aimed at defectors leads to higher cooperation rates despite the fact that there is punishment mistakenly aimed at cooperators. Under both CDRs, too few defectors were punished to enforce cooperation. Yet, under the IDR resources were wasted on punishments mistakenly aimed at cooperators, and hence in the end also the IDR is unable to maintain profits above baseline levels. Grechenig et al. [27], Fischer et al. [28], Ambrus and Greiner [41], Bornstein and Weisel [51], and Patel et al. [52] find similar results for related settings.

Our results stand in stark contrast to a study conducted by Ambrus and Greiner [53], who study a similar research question in a slightly different experimental setup. Unlike us, they find a significantly positive effect of a CDR on cooperation both with and without noise. Our experiment differs from theirs in five important points: (1) Ambrus and Greiner [53] use a five-person PD, and the same group members interact with each other in each round. We use a six-person PD, and groups are disbanded and randomly formed anew after each round. (2) They use a noise level of 10%, whereas in our case 20% of the contribution decisions are noisy. (3) In their experiment noise distorts the decisions of cooperators while defectors’ decisions are observed perfectly. In our case noise works in both directions, also distorting defectors’ decisions. (4) In their setup, the contribution signal is noisy; all group members observe the same contribution decision of the other group members. In our case the *perception* of the contribution signal is noisy; each group member can observe another group member’s contribution decision differently due to noise. (5) In their CDR, all group members incur a cost if punishment is implemented. In our case only those who voted incur a punishment cost if punishment is implemented.

It seems plausible that the smaller group size, the repeated game setting, and the smaller noise level make the experimental setup of Ambrus and Greiner [53] more favorable to the emergence of cooperation than our experiment (also see [56]). Also, the fact that defection in their study is always observed accurately makes it easier to coordinate on pro-social punishment under a CDR compared to our setting, where pro-social punishment levels fall sharply under CDRs with noise. We thus believe that this will explain the difference in our two studies’ results. It may be therefore fruitful to narrow down the parameter space within these factors in order to pinpoint the conditions under which CDRs promote cooperation and conditions in which they do not if public monitoring is noisy.

It is interesting to note that in both studies, in conditions without noise an IDR fails to maintain high cooperation rates over time. This contradicts numerous previous findings (e.g., [20,21]). This finding may be due to the fact that each punishment reduced recipient payoffs by six points and thus did not sufficiently discouraged defection. We also find that subjects become more willing to cooperate without noise as more group members are required to agree on punishment decisions. Indeed, most antisocial punishments are ruled out while most prosocial punishments are implemented. This suggests that both decision rules were tailored to the proportion of prosocial and antisocial punishers in the population. Conversely, with noise cooperation and earnings were not increased by implementing punishment through CDRs. Prosocial punishment rates were low under CDRs with noise because prosocial punishment did not reach enough consensus. Despite their consensual nature and ability to prevent anti-social punishment, collective decision rules for implementing peer punishment do not seem to be always effective in maintaining cooperation if cooperation decisions cannot be perfectly observed.

## Supporting information

S1 Data (DTA)

S1 Additional analyses and study material (PDF)
