# Supplementary material for: Consensual punishment does not promote cooperation in the six-person prisoner's dilemma game with noisy public monitoring
Source: PLoS One. 2017 Nov 27;12(11):e0188503. doi: 10.1371/journal.pone.0188503 (PMC5703489; doi:10.1371/journal.pone.0188503)
Supplement: S1 Additional analyses and study material — (PDF) [file pone.0188503.s002.pdf]

S1 Additional analyses and study material

Consensual punishment does not promote cooperation in the six-person prisoner's dilemma game with noisy public monitoring

Nynke van Miltenburg[1], Wojtek Przepiorka[1*], Vincent Buskens[1]

[1] Department of Sociology / ICS, Utrecht University, Utrecht, The Netherlands

[*] Corresponding author
E-mail: w.przepiorka@uu.nl (WP)

Order effects, descriptive table, regression models

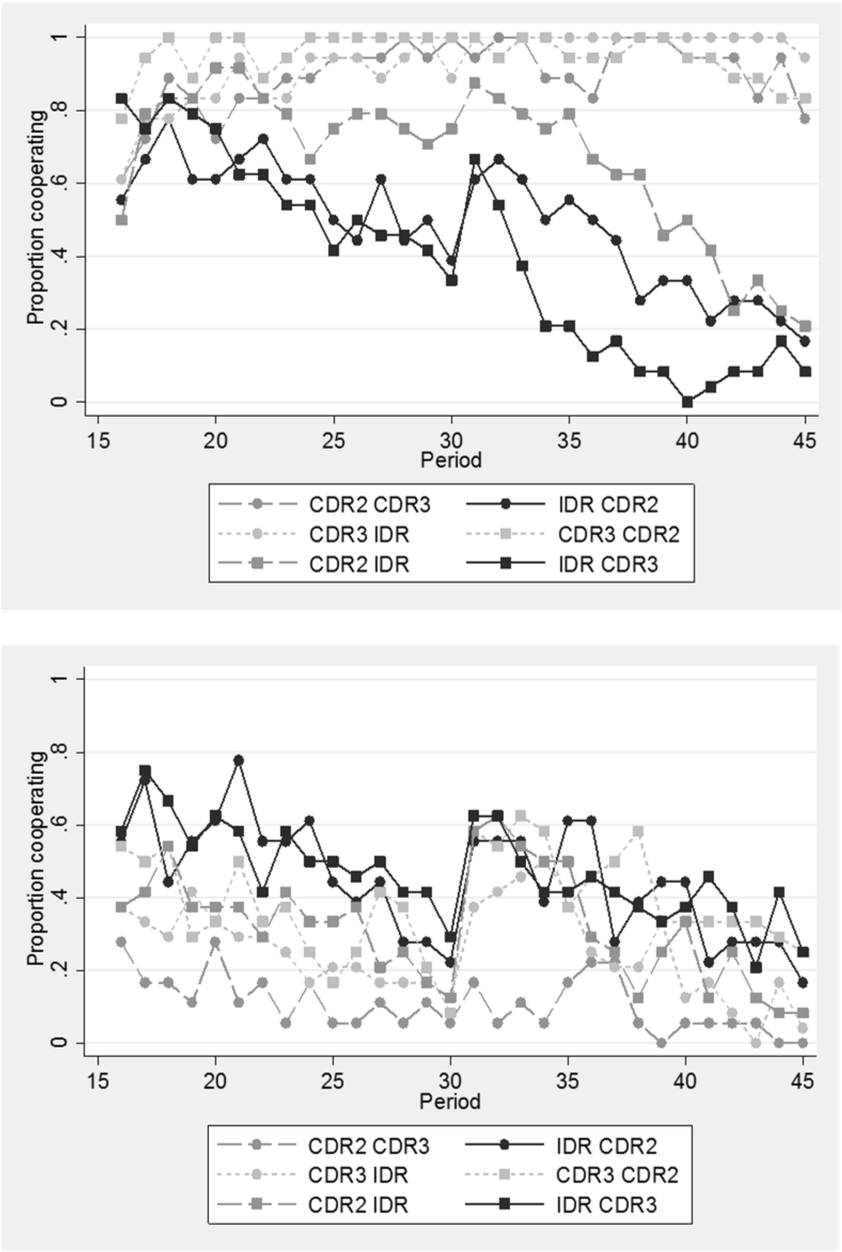

Fig A: Cooperation rates in each period of the first and second punishment sequence, separated by sessions, without (top) and with (bottom) noise. Note that the change in experimental condition occurs after period 30 (dashed line).

Table A: Average cooperation, earnings (excluding punishment endowment), and punishment of observed defectors in each experimental condition, in the baseline and first punishment sequence (7,955 punishment decisions, 7,560 PDs, 252 subjects).

	Cooperation		Earnings		Punishment if target is obs. defector	
	Mean	Sd.	Mean	Sd.	Mean	Sd.
Baseline no noise	0.225	0.418	26.300	9.139		
Baseline noise	0.269	0.444	27.537	9.649		
IDR no noise	0.587	0.493	29.498	9.799	0.375	0.484
IDR noise	0.511	0.500	25.841	10.716	0.380	0.485
CDR2 no noise	0.814	0.389	38.229	8.790	0.621	0.486
CDR2 noise	0.244	0.430	25.029	9.128	0.146	0.354
CDR3 no noise	0.915	0.279	43.630	6.549	0.613	0.488
CDR3 noise	0.299	0.458	26.128	10.470	0.280	0.449

Table B: Logistic regression on the decision whether or not to cooperate and on the decision whether or not to punish an observed defector, and linear regression on period earnings (excluding punishment endowment), all with decisions nested in subjects and sessions, in the baseline and first punishment sequence (7,955 punishment decisions, 7,560 PDs, 252 subjects).

	Cooperation		Earnings		Punishment obs. defectors	
	Coeff.	S.e.	Coeff.	S.e.	Coeff.	S.e.
Noise	0.098	0.223	1.146	0.635	0.286	0.672
IDR	1.937**	0.141	2.928**	0.492		
× noise	-0.520**	0.198	-4.639**	0.696		
CDR2	3.573**	0.177	12.563**	0.492	2.235**	0.688
× noise	-3.816**	0.233	-15.205**	0.696	-5.010**	0.987
CDR3	4.306**	0.225	16.906**	0.529	2.466**	0.752
× noise	-4.081**	0.262	-18.185**	0.703	-3.707**	1.004
Constant	-1.572**	0.159	26.333**	0.454	-1.054*	0.480
σu	0.130	0.217	0.817	0.267	0.291	0.359
σe	1.459	0.087	2.399	0.162	2.649	0.179
Log Likelihood	-3600.076		-27544.172		-3114.541	

*Significant at .05-level; ** Significant at .01-level (2-sided)

Table C: Logistic regression on the decision whether or not to cooperate and on the decision whether or not to punish an observed defector, and a linear regression on period earnings (excluding punishment endowment) in the baseline and first punishment sequence (7,955 punishment decisions, 7,560 PDs, 252 subjects). All models are estimated with robust standard errors accounting for session-level clustering by means of clustered sandwich estimator.

	Cooperation		Earnings		Punishment obs. defectors	
	Coeff.	S.e.	Coeff.	S.e.	Coeff.	S.e.
Noise	0.238	0.169	1.237	0.868	0.018	0.236
IDR	1.590**	0.138	3.198*	1.444		
× noise	-0.546**	0.164	-4.895**	1.540		
CDR2	2.715**	0.203	11.929**	0.942	1.001*	0.408
× noise	-2.845**	0.419	-14.437**	1.623	-2.274**	0.477
CDR3	3.611**	0.570	17.330**	2.173	0.969**	0.324
× noise	-3.466**	0.581	-18.739**	2.208	-1.422**	0.376
Constant	-1.237**	0.135	26.300**	0.657	-0.509*	0.232
Log likelihood/ R^2		-4225.501		0.238		-4603.520

*Significant at .05-level; ** Significant at .01-level (2-sided)

Table D: Logistic regression on the decision whether or not to cooperate and on the decision whether or not to punish an observed defector, and a linear regression on period earnings (excluding punishment endowment) in the baseline and first punishment sequence (7,955 punishment decisions, 7,560 PDs, 252 subjects). All models are estimated with robust standard errors accounting for session-level clustering by means of bootstrap estimation (based on at least 1100 successful replications).

	Cooperation		Earnings		Punishment obs. defectors	
	Coeff.	S.e.	Coeff.	S.e.	Coeff.	S.e.
Noise	0.238	0.162	1.237	0.832	0.018	0.253
IDR	1.590**	0.132	3.198*	1.548		
× noise	-0.546**	0.159	-4.895**	1.650		
CDR2	2.715**	0.237	11.929**	1.032	1.001	0.543
× noise	-2.845**	0.512	-14.437**	1.759	-2.274**	0.611
CDR3	3.611**	0.670	17.330**	2.223	0.969*	0.486
× noise	-3.466**	0.673	-18.739**	2.265	-1.422**	0.527
Constant	-1.237**	0.129	26.300**	0.619	-0.509*	0.249
Log likelihood/ R^2		-4225.501		0.238		-4603.520

*Significant at .05-level; ** Significant at .01-level (2-sided)

Table E: Logistic regression on the decision whether to cooperate in period t in the first punishment sequence (3,528 PDs, 252 subjects). Both models are estimated with robust standard errors accounting for session-level clustering. In model M1 the clustered sandwich estimator is used and in M2 bootstrap estimation is used. The latter is based on 221 successful replications.

	M1		M2	
	Coeff.	S.e.	Coeff.	S.e.
Noise	0. 797*	0.391	0. 797	0.484
CDR2	0.731**	0.108	0.731**	0.156
× noise	-1.172**	0.234	-1.172**	0.339
CDR 3	1.357**	0.341	1.357**	0.451
× noise	-1.687**	0.346	-1.687**	0.453
Own contribution $t-1$	3.598**	0.420	3.598**	0.484
× noise	-1.210*	0.474	-1.210*	0.520
Punished while defecting $t-1$	1.239**	0.339	1.239*	0.488
× noise	-0.661	0.395	-0.661	0.528
Punished while cooperating $t-1$	-0.771**	0.148	-0.771**	0.186
× noise	0.890*	0.186	0.890**	0.226
Obs. N other cooperators $t-1$	0.245**	0.051	0.245**	0.052
× noise	-0.031	0.076	-0.031	0.074
Period	-0.031	0.020	-0.031	0.021
× noise	-0.035	0.021	-0.035	0.022
Constant	-2.649**	0.407	-2.649**	0.458
Log likelihood		-1520.216		-1520.216

*Significant at .05-level; ** Significant at .01-level (2-sided)

Experimental instructions

This is the English version of the experimental instructions. Subjects could choose for a Dutch or an English version of these instructions. The instructions for part 2 were handed out after part 1 was finished. Italics indicate parts of the instructions that were only displayed in the sessions with noise.

# - Instructions -

Welcome

Please read the following instructions carefully. These instructions are the same for all participants. The instructions state everything you need to know in order to participate in the experiment. If you have any questions, please raise your hand. One of the experimenters will approach you and answer your question.

You can earn money by means of earning points during the experiment. The number of points that you earn depends on your own choices and the choices of other participants. At the end of the experiment, the total number of points that you earned will be exchanged at an exchange rate of:

160 points = 1 Euro

The money you earn will be paid out in cash at the end of the experiment. Other participants will not see how much you have earned. During the experiment you are not allowed to communicate with other participants. Please turn off your mobile phone and put it in your bag. You may only use the functions on the screen that are necessary to carry out the experiment. Thank you very much.

The decision situation

First, we introduce the decision situation in which you will interact. You will learn about the procedure of the experiment later.

You are a member of a group of **six participants**. You and the five other members of your group are endowed with **20 points** each. You must all choose whether you want to keep your

points to yourself in a **private account** or contribute your points to the **group account**. You can only contribute the **entire** amount of 20 points to the group account, or keep the whole endowment to yourself and contribute 0 points.

When all members of your group have decided on their contribution to the group account, the total number of points in the group account is **multiplied by 2.4**. The multiplied amount is then **equally** distributed among all six group members. When you have kept your points in your private account, your earnings from the group account will be added to these points. This holds for each participant in your group.

Table 1 below shows how many points you earn, given the choices you make and the number of others in your group who contribute to the group account. For example, suppose you contribute your 20 points to the group account, and four of the others contribute as well. The total number of people who contribute, including yourself, is five. The sixth group member keeps his/her points on the private account. Table 1 shows that you then earn 40 points. You can verify this yourself: the total contribution to the group account is 5×20 = 100 points. Multiplied by 2.4 gives 240 points in the group account, divided by six group members is 40 points each. Note that Table 1 also applies to the other group members. For the group member who contributed 0 points in the example, five others (including you) have contributed. This group member then receives the 40 points from the group account in addition to the 20 points on his/her private account, and earns 60 points. You can see this in Table 1.

Table 1: the number of points you earn for different own contributions and number of other group members who contribute to the group account.

Your contribution	Number of other group members who contribute 20 points					
	0	1	2	3	4	5
0	20	28	36	44	52	60
20	8	16	24	32	40	48

Before you continue reading the instructions, please choose the language in which you want to do the experiment and click "OK" on your computer screen. You will proceed to a number of control questions. Answer the questions to check whether you have understood the decision situation. Your answers to the control questions have no influence on your payment.

The Experiment

This experiment consists of **three parts**. Each part includes the decision situation described above. All three parts are series of 15 decision rounds. These instructions are for part 1; the instructions for part 2 and for part 3 will follow later.

Instructions part 1

You will now participate in the first series of **15 decision rounds**. You are randomly matched to five other participants. In each round, you receive the endowment of 20 points and decide whether you contribute these points to the group account. This is the only decision you have to make in every round, and will determine your earnings. After this decision you are again randomly matched to five other participants.

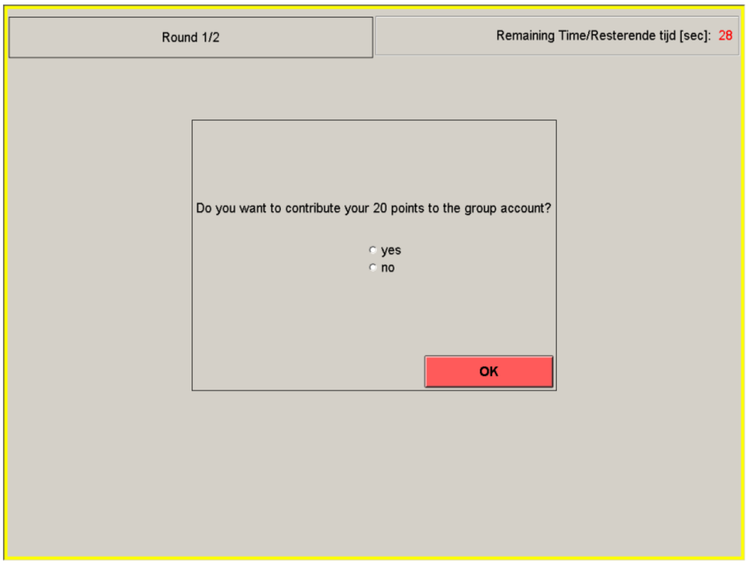

You will see a screen like the one shown above. You click whether or not you want to contribute your 20 points to the group account. If you choose "no", the 20 points will go to your private account. Once you have made a choice, click the "OK" button to confirm your decision.

After all participants have made their decision for the round, the computer will calculate the payoffs and tabulate the results. You will be informed about the contribution every member of your group has made to the group account.

***Please note: this information is not always correct**. For every contribution that you observe, there is a **20%** chance that this is not the real contribution that this group member made. This means that, for each contribution of 20 that you see, there is a 20% chance that in reality this group member did not contribute to the group account. Likewise, for every contribution of 0 that you see, there is a 20% chance that this group member did contribute. Whether you see the correct contribution will be determined **independently** for every contribution that you see. It can happen that one, several, or none of the contributions that you see are displayed incorrectly.*

*The 20% chance to see a wrong contribution also holds for the others in your group. Again, this chance is independent for every other group member. For example, every group member has a 20% chance to wrongly observe the contribution of the person who is displayed for you as "Number 6". It can happen that for one, several, or none of the other group members the contribution of this person is wrongly displayed. **This also means that every other group member has a 20% chance to see your contribution incorrectly**. One, several, or none of the other group members can observe your contribution wrongly.*

*You will not be informed which of the contributions that you observed were displayed incorrectly. However, your earnings will be determined by the **real** contributions of all group members. At the end of every round you are informed about the number of points that you earn in this round if the contributions that you see are the actual contributions. Please note that these might not be your real earnings.*

This procedure is repeated for 15 rounds. The participants with whom you are together in your group will be **randomly selected after every round**. You will not be told each other's identities at any time during or after the experiment.

When you have finished the first part of the experiment you will receive new instructions for part 2 and finally for part 3.

Instructions Part 2

These instructions are for the second series of 15 decision rounds. The decision situation as explained in the first instructions remains unchanged. You will thus in every round be asked whether you want to contribute your endowment of 20 points to the group account. However, every round now has a second part that follows after every member of your group has decided

whether they want to contribute. In this second part all group members can **decrease** the earnings of the others. To do this, **every round** again you receive an **additional endowment of 10 points**. You will see the following screen:

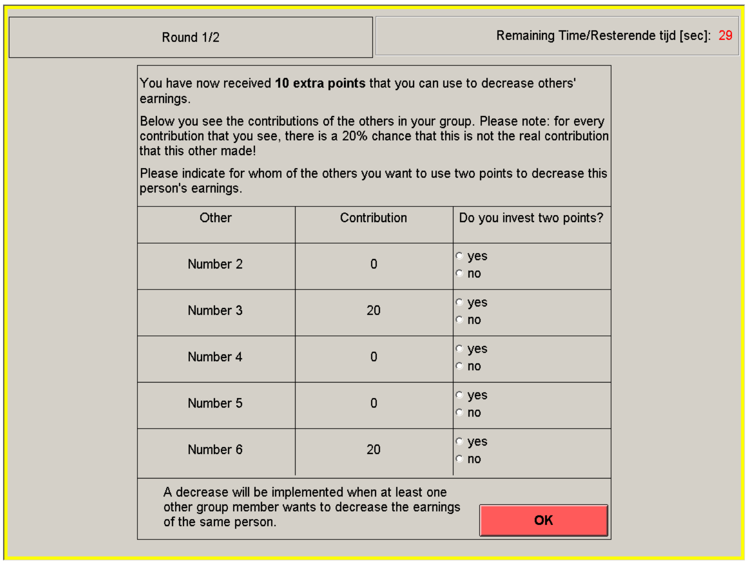

In the second column you see the contribution every other member of your group ("Number 2" to "Number 6") has made to the group account. ***Please note****: again, there is a* ***20% chance****, for every contribution that you see here, that this is not the real contribution of this group member. This chance is again independent for everyone, just like in part 1 of the experiment. If you do not remember exactly what this implies, have another look at part 1 of the instructions.*

In the third column, you decide whether or not you want to decrease the earnings of each other group member by clicking "yes" or "no". You make this decision for each group member separately. If you decide that you want to decrease the earnings of another member, this is only possible **if at least one of the other members of your group also wants to decrease this person's earnings**.

When enough people want to decrease the points of the same group member, for example if you and "Number 2" both want to decrease the earnings of "Number 4", you and "Number 2" will lose **2 points** from the 10 extra points that you have both received in this second phase. "Number 4" will lose **6 points** for every group member who decreased his/her earnings, so in this case 2×6 = 12 points. If there are no two people who want to decrease the earnings of the

same person, for example only “Number 2” wanted to decrease the earnings of “Number 4”, the decrease will not be executed and both “Number 2” and “Number 4” will keep their points.

Your total profit for the round will be the sum of your earnings from part 1 and part 2 of that round. The part of the additional endowment of 10 points that you do not spend on decreasing others’ payoff will be added to your earnings. At the end of the round, you will again see a screen showing how much the others have contributed, and with how many points their earnings were decreased. *You will see the same contributions here as you saw in the screen where you could choose whether or not to decrease others’ points.* ***Contributions you observed incorrectly in that screen, will thus be displayed incorrectly again****. Hence, you will never be informed about the true contributions. However, your earnings will be determined by the* ***real*** *contributions of all group members. At the end of every round you are informed about the number of points that you earn in this round if the contributions that you see are the actual contributions. Please note that these might not be your real earnings.*

If only one of the others wanted to decrease your earnings, this will NOT be shown to you. Similarly, if you wanted to decrease the earning of another group member, but not at least one other group member wanted this as well, this will NOT be shown to the group member whose earnings you wanted to decrease. If at least two of the others decided to decrease your points, you will see on this screen that your points have been decreased, but you will NOT see which participant paid to have your earnings decreased. Similarly, if you and one or more other group members decide to decrease the points of another group member, (s)he will be shown that his/her points are decreased, but (s)he will NOT be informed that you are one of the persons who paid to decrease his/her points.

This procedure is repeated for 15 rounds. The participants with whom you are together in your group are again **randomly selected after every round**. “Number 2”, to “Number 6” will thus most likely be a different person than in the previous round. You will not be told each other’s identities at any time during or after the experiment. After this second series you will receive new instructions for the third series.
